# Manipulation of Self-Expansion Alters Responses to Attractive Alternative Partners

**DOI:** 10.3389/fpsyg.2020.00938

**Published:** 2020-05-26

**Authors:** Irene Tsapelas, Lane Beckes, Arthur Aron

**Affiliations:** ^1^Department of Psychology, Stony Brook University, Stony Brook, NY, United States; ^2^Department of Psychology, Bradley University, Peoria, IL, United States

**Keywords:** close relationship, attention to alternatives, self-expansion, romantic love, social neuroscience

## Abstract

Past behavioral research has examined relationship infidelity as a potential outcome of focusing on attractive alternative partners when already in a relationship. The extent to which individuals find such alternatives attractive has been shown to be associated with various factors in the relationship, including self-expansion. However, no previous research has tested the role of self-expansion experimentally. This paper presents two experiments that directly manipulate self-expansion to determine the effect of self-expansion on responses to attractive alternative partners. Participants primed to experience a higher need for self-expansion had better memory for attractive alternatives with self-expanding traits dissimilar to their partner’s versus attractive alternatives with self-expanding traits similar to their partner’s. Additionally, participants primed with self-expansion (via a video of their partner discussing ways in which life with one another is exciting, novel, and challenging), had less fMRI BOLD response to attractive alternatives of the opposite sex in regions associated with perception of attractive faces (anterior cingulate and medial prefrontal cortex) relative to when they were primed with love (via a video of their partner discussing times they felt strong feelings of love for one another), or neutral content (via a video of their partner discussing some times in which they engage in mundane, routine activities together). The magnitude of this effect in the ACC correlated with relationship closeness as measured by the inclusion of the other in the self scale.

## Manipulation of Self-Expansion Alters Responses to Attractive Alternative Partners

Focusing on attractive alternative partners when one is already in an established pair bond can lead to several negative outcomes, including infidelity and relationship dissolution (for a review of the infidelity literature, see Tsapelas et al., 2011). The extent to which individuals attend to and remember such alternatives, however, may be affected by various factors within the relationship, including love for the partner and self-expansion in the relationship. In the last 30 years, researchers from a wide array of disciplines have studied various correlates and predictors of infidelity, including individual difference and demographic variables (e.g., attachment style, gender), and characteristics involving the primary relationship (e.g., love, satisfaction, commitment). Further, the underlying processes involved in infidelity have been approached from a variety of theoretical perspectives, including evolutionary, attachment, and investment theories, and, most recently, self-expansion model.

## Introduction

### Self-Expansion Model

Aron and Aron’s (1986) self-expansion model of close relationships posits that people are motivated to enter relationships in order to enhance the self and increase self-efficacy. The main way that people seek to expand the self in the context of relationships is by “including others in the self” (IOS). Over time, the other’s resources, perspectives, and identities are integrated into one’s own self-concept. These principles have received considerable research support and have been applied to the study of various relationship issues, including romantic love, intergroup relations, breaking up, and relationship boredom (for a review, see Aron et al., 2013).

The self-expansion model suggests that in the beginning phase of a relationship when forming a pair bond (especially rapidly coming to include the partner in the self) is highly self-expanding. This rapid self-expansion is associated with feelings of great pleasure, arousal, and excitement. As time passes, the relationship becomes more predictable and there can be a decline of self-expansion. This decline in self-expansion may be a key factor in the typical decline in relationship satisfaction over time (e.g., Bradbury et al., 2000). Empirical work indicates that a loss of excitement may be a major driving force behind declining relationship quality (e.g., Tsapelas et al., 2009). Because alternative partners’ offer novelty, new opportunities for self-expansion, and excitement, declines in self-expansion may be an important contributor to relationship infidelity.

In a sample of dating college students Lewandowski and Ackerman (2006) found that self-reported self-expansion variables (current and potential self-expansion from the relationship and inclusion of the partner in the self) accounted for a large portion of the variance in self-reported susceptibility to infidelity (i.e. likelihood that participants would engage in various infidelity behaviors). Further, VanderDrift et al. (2007) found that reported lack of relationship-derived self-expansion increases attention to alternatives, and decreases devaluation of alternatives. A combination that is likely to promote infidelity.

Other work (Le et al., 2009) examined the relative strength of relationship closeness (IOS) and self-expansion opportunities in predicting sexual infidelity. In a sample of college student participants, self-expansion but not closeness, significantly predicted less sexual infidelity. The same results were found in a second study of college students over a 4-week winter break. This work highlights a distinction between the process of self-expansion and the state of self-other inclusion.

The distinction between self-expansion and inclusion of the other in the self is most clear as a distinction between process, outcome, and the emotional content of each. Self-expansion refers to the process by which one develops new aspects of the self through the relationship. Critically, learning of this type will be dependent on the mesocorticolimbic dopamine system (see Adcock et al., 2006), which is central to predictive reward and motivation (Berridge, 2012). Moreover, greater activity in this dopamine system is associated with romantic love (Fisher and Brown, 2002) and feelings of excitement and desire. Because self-expansion theoretically involves learning, motivation, and reward, self-expansion is a hot process that contributes to intense emotion and promotes attraction to and desire for the partner.

Inclusion of the other in the self, alternatively, is about relationship closeness and is often an outcome of the interpersonal interactions driven by self-expansion. Inclusion of the other in the self is much more strongly related to already formed memories, habits, and patterns of living. A close relationship partner becomes integrated into the way an individual regulates their own need and desires (Saxbe et al., 2019), forming a regulatory system that is dyadic rather than individual in nature. Within this system the individual includes the partner in their planning and resources. Via this cognitive organization, the partner is integrated in person’s cognitive systems such that they are included in the person’s self-concept. This type of self-other integration is typically cooler once completed and will involve much less emotional content as long as the dyad is relatively predictable and maintains stability. Thus, inclusion of the other in the self is likely a commitment magnifying phenomenon, but relative to self-expansion is a cool psychological process, and therefore not the same as hot processes like self-expansion and romantic love.

### Romantic Love and Attention to Alternative Partners

Romantic love also seems to reduce attention to alternatives, and perhaps deters infidelity. Maner et al. (2008) found that priming thoughts and feelings of romantic love for one’s current partner reduced attention to photos of physically attractive alternatives in a visual cueing measure. In this study, participants were assigned to either a romantic love condition (in which they wrote a brief essay about a time they experienced strong feelings of love for their current partner) or a control condition (in which they wrote about a time they felt extremely happy). After writing the essay, participants completed a version of the visual dot-probe procedure which assessed how efficiently they were able to shift their attention away from one stimulus location to another. Participants primed for romantic love (compared to those in the control condition) demonstrated less visual attention to the photos of attractive alternative partners.

A later study (Maner et al., 2009) used the same visual cueing task with two different implicit manipulations intended to prime mating: In study 1, participants were primed with words highly relevant to mating (e.g., kiss, lust) and in study 2, participants completed a sentence unscrambling task with words highly relevant to mating. Single participants responded to the mating primes by increasing attention to physically attractive alternatives, but participants in a committed romantic relationship were inattentive to those alternatives. Another study (Gonzaga et al., 2008) found that romantic love (but not sexual desire) led participants to display poorer memory for characteristics of an attractive alternative, specifically attractiveness-related details (e.g., fitness and beauty cues) but not attractiveness-irrelevant details of the alternative. Further, romantic love, but not sexual desire, predicted greater commitment to the current partner.

Perceiving and focusing on desirable alternatives weakens relationship satisfaction and stability, so individuals who are motivated to maintain their relationships will either be inattentive toward alternatives and/or perceive alternatives as less desirable. In contrast, partners lower in love may be more likely to attend to and be attracted to alternatives. Theoretically, self-expansion through a relationship partner may be critical to promoting derogation and decreased attention to alternative partners, but to our knowledge no experimental work has demonstrated a causal effect of self-expansion on attention to alternatives.

### The Present Research

Recent behavioral research has indicated that self-expansion and IOS (inclusion of other in the self) play an important role in the perception and evaluation of attractive alternatives. However, this work has entirely been correlational. The present research expands this work in several key ways. Most importantly, no previous research has studied the role of self-expansion experimentally. Both of the present studies specifically manipulate self-expansion (and do so, in two different ways). No previous studies have examined the prediction from the self-expansion model that under conditions of general high self-expansion need, potential alternative partners would be especially salient who have desirable characteristics (which could thus be included in the self if one had a relationship) that the current partner does not have. This is the focus of Experiment 1. Finally, no previous research has examined the role of self-expansion on attention or attraction to potential alternatives. Experiment 2 does so via neuroimaging, which can help distinguish whether self-expansion simply decreases attention to alternatives or whether it decreases actual attraction via the activity of dopaminergic systems. This is a key contribution of Experiment 2. Experiment 2 is also pioneering in that (a) it manipulates degree of self-expansion in the relationship and (b) manipulates degree of relationship love as a comparison condition.

## Experiment 1

Research from the self-expansion model indicates that if one’s primary relationship is not meeting self-expansion needs, individuals may look outside the relationship to fulfill these needs. Specifically, the model predicts that if one is feeling that one’s self-expansion needs are not being met, the most desirable alternatives would be ones who possess traits one’s long-term partner does not. In contrast, if one is feeling adequate self-expansion, and circumstances (such as opportunity) led to an interest in alternatives, the most desirable would be ones that are actually possessed by one’s current partner. In this case, one will presumably not need additional and varied forms of self-expansion from a potential alternative, and will instead prefer traits representative of his or her partner.

This experiment is the first of which we are aware to directly investigate how general self-expansion needs influence the way people process information about specific types of alternative partners. We hypothesized that a primed need for self-expansion in one’s life will predict greater attention to, and memory for, attractive alternatives that possess self-expanding traits the partner does not have (versus attractive alternatives with self-expanding traits the partner does have).

## Method

As part of an on-line “mass-testing” session, participants in a current relationship rated various traits for the degree they were possessed by their partner and for how desirable those traits are in general in a romantic partner. At a subsequent, supposedly unrelated, laboratory session, participants were primed with either high or low need for self-expansion, then participated in a task designed to assess memory for and attention to several potential attractive relationship alternatives, some of whom had desirable traits possessed by their current partner, and some with desirable traits not possessed by their partner. Thus, the design was a 2 (high vs. low primed self-expansion need) × 2 (partner-similar versus partner-dissimilar traits) between-subjects design.

### Participants

149 participants (111 women, 38 men) recruited from the Stony Brook University Psychology Department subject pool received course credit for their participation. All participants were in a committed, exclusive relationship of at least 6 months; mean age, 19.76; mean relationship length, 22.91 months; 87.9% exclusively dating; the remainder were either married or engaged.

### Partner Attributes

In the initial online mass-testing session, participants rated 48 desirable and potentially self-expanding traits, first for how representative each was of their current partner, and second for how desirable each trait would be in a potential romantic partner. These measures were separated by a substantial number of other questionnaires, which reduced potential carryover effects. Example traits included “ambitious,” “funny,” “talented,” “sensitive,” “creative,” “musical,” and “intelligent”–traits rated high in general “likeability” in previous research (Anderson, 1968). The questionnaires completed between the two focal ratings included a number of short-form versions of standard relationship measures.

Before the lab session, for each participant, attributes the participant had rated as highly desirable in a general partner (>7 on 1–10 scale) were used to create 10 target pairings, each trait with a photo of an attractive, opposite-sex face. There were 10 photos total–5 associated with traits the participant had rated characteristic of the partner; 5 with traits the participant had rated as not characteristic of the partner. The 10 attractive, opposite-sex faces were adapted from past research (e.g., Maner et al., 2008, 2009). Mean attractiveness ratings (1–7 scale) were 4.16 for female photos, and 4.06 for male.

Thus, photos of attractive, opposite-sex faces were each paired with an attribute purportedly describing the alternative, designed by the researchers to reflect either (a) potentially self-expanding attributes rated as highly desirable that the participant’s actual partner possesses or (b) potentially self-expanding attributes rated as highly desirable that the participant’s actual partner does not possess. Traits of each type were randomly paired with photos, separately for each subject. No differences in mean desirability were found for partner-similar vs. partner-dissimilar traits.

### Procedures

In the lab session (approximately 2–3 weeks after mass-testing) participants were randomly assigned to either a high or low self-expansion need condition (i.e. how much self-expansion one feels they are experiencing in life in general) employing a priming manipulation used in prior research (see Wright et al., 2004). First, they completed a short self-description and bogus personality test. In the high self-expansion need condition, participants were told that their responses to the personality test indicated that their life was rather predictable and stagnant – that they were in a bit of a “rut,” and they demonstrated concern they were not getting the resources needed to meet potential upcoming challenges. In the low self-expansion need condition, participants were told that the test indicated they had recently experienced considerable psychological change, they were somewhat overwhelmed with the number of new things they were trying to manage in their life, and they probably needed time to sort out these changes.

Next, participants were asked to take part in a memory task, as part of an experiment ostensibly having nothing to do with relationships, alternatives, or their own relationship. They viewed photos of 10 attractive, opposite-sex faces, each paired with a trait. Prior to viewing the trait/photo pairs participants were told that they would take part in a subsequent memory test so they should try to remember as many of the photo-attribute pairings as possible. Participants then completed a dot-probe computer task used to measure visual attention to attractive alternatives (for a complete description, see Maner et al., 2008 or Maner et al., 2009). Finally, following the attention task, approximately 12 min after initially encoding the photo/trait pairs, participants were tested on their recall for the attribute-photo pairings by viewing each of the 10 photos (10 s each) on a computer screen and listing (on paper) the associated trait for each photo. The dependent variable was the number of correctly recalled traits (out of a possible 5) for each target type (partner-similar, partner-dissimilar). (At debriefing, no participant identified the experiment’s true purpose or its relation to earlier ratings).

## Results

**FIGURE 1:**
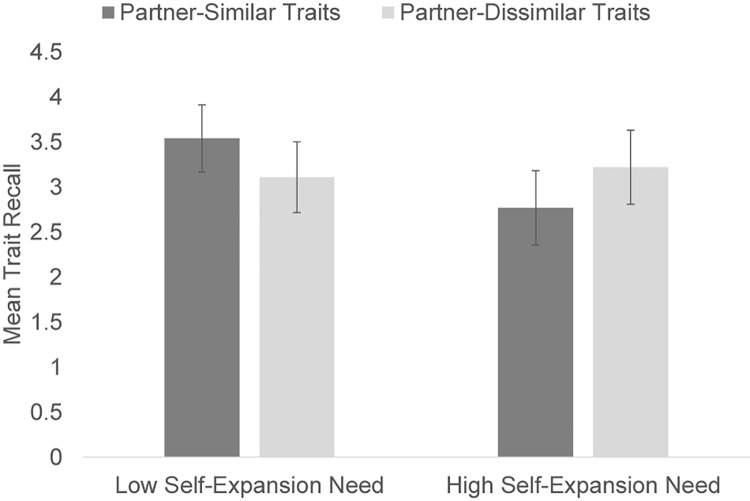
Depicts the mean recall for partner-similar and partner-dissimilar traits in the high and low self-expansion need conditions with 95% confidence intervals.

To test the key research questions, we employed a 2 × 2 × 2 mixed-design ANOVA with self-expansion need (high vs. low; between subjects), gender, and partner-similar versus partner-dissimilar traits (within-subjects variable) as factors. The condition × partner trait interaction was significant, *F*(1, 145) = 12.04, *p* = 0.001, η^2^*_P_* = 0.08. As shown in Figure 1, participants primed for high self-expansion need, *t*(73) = 2.05, *p* = 0.04, correctly recalled more partner dissimilar (*M* = 3.22, SE = 0.21) than partner similar traits (*M* = 2.77, SE = 0.20); those primed with low need, *t*(75) = −3.02, *p* = 0.004, recalled more partner similar (*M* = 3.54, SE = 0.19) than dissimilar traits (*M* = 3.11, SE = 0.21). No other main or interaction effects (including those for gender) were significant.

We also conducted a similar analysis with dot-probe response-time as the focal DV. No main or interaction effects approached significance.

## Experiment 1 Discussion

This was the first study of which we are aware to examine self-expansion needs on the way people process information about alternative relationship partners. When experiencing high need for self-expansion, people displayed better memory for potential alternatives that have desirable traits their partner does not possess. In contrast, those experiencing sufficient self-expansion in life, have better memory for potential alternatives that have desirable traits their partner does possess.

This work suggests a central role for an individuals’ state degree of self-expansion motivation in relationship cognition, particularly cognitive processes involving the perception of alternative partners, and perhaps even infidelity. If self-expansion needs are not being met, an alternative’s potentially self-expanding traits may be more appealing than such traits of the partner (which are presumably already contributing to the individual’s self-expansion); the alternative may be attractive due to the new and varied forms of self-expansion (and new traits to include in the self) that he or she can offer. However, when self-expansion needs are already being met, individuals may not feel it necessary to look outside the relationship for additional self-expansion opportunities, and may focus more on the self-expanding attributes of the partner. Or if situations promote an interest in alternatives, they are likely to be alternatives like the partner one already has.

Another way to interpret these results is that partner’s traits matter most: If one needs more self-expansion, it may seem that the traits one’s partner has do not add much to life; but if one’s life is highly self-expanding, if the possibility of alternatives arises, one may want more of what is already working.

At the same time, a limitation of the present findings, is that we did not find parallel results using a standard attentional task that has proven successful in several previous studies of interest in potential alternatives (e.g., Maner et al., 2008). That is, although the predicted pattern was found for memory, it was not significant for attention. This may indicate that attractive alternatives do capture earlier attention, but later processes involved in sustained attention, encoding, and evaluation are modified by self-expansion in the current relationship. If self-expansion is high, individuals may avoid encoding information regarding alternatives as less important because their needs are well served in their current relationship. Such a pattern makes sense if desire for the current partner suppresses desire for alternatives. In animal models there is evidence that increasing the incentive salience of one target can diminish the incentive salience of other targets (DiFeliceantonio and Berridge, 2016) as measured through behavior and the activity of dopaminergic systems in the brain. Increased incentive salience should improve memory encoding and attraction. Fortunately, there are well known neural systems involved in incentive processes, making exploring this question possible using function magnetic resonance imaging (fMRI).

## Experiment 2

Experiment 2, sought to investigate the role of self-expansion (and love) in limiting attraction to alternatives at the neural level by investigating incentive responses to attractive alternatives in dopaminergic systems after self-expansion, romantic love, and neutral primes. Given that self-expansion and romantic love are theoretically linked to processes mediated by the mesocorticolimbic system we should see changes in blood oxygenation level dependent (BOLD) response to attractive alternatives in this system when self-expansion and romantic love with the partner are manipulated, Thus, the goal of this study is to determine whether self-expansion and romantic love manipulations modify reward related responses in the mesocorticolimbic system to attractive alternatives. Having a partner who promotes self-expansion and elicits strong feelings of romantic love should be associated with higher incentive salience for the partner (i.e. predictive reward; see Berridge, 2012 for a discussion of incentive salience and the mesocorticolimbic system). Moreover, increasing the incentive salience of a partner should reduce the relative incentive salience of alternative partners through cortical dopaminergic systems (DiFeliceantonio and Berridge, 2016). If self-expansion reduces attention to attractive alternatives, we should see evidence of reduced activity in mesocorticolimbic activation when self-expansion with one’s partner is increased.

Interpersonal attraction and romantic love are strongly related to activity in the mesocorticolimbic dopamine system (Fisher and Brown, 2002) which is crtical for learning, motivation, and incentive salience. Much of the neuroimaging work on interpersonal attraction has focused on facial attractiveness. Perceiving a highly attractive face is associated with increased BOLD response in various subcortical and medial prefrontal regions known to be innervated by ventral dopamine pathway (O’Doherty et al., 2003; Winston et al., 2007). A recent meta-analysis (Mende-Siedlecki et al., 2013), indicates that a portion of this pathway including the medial prefrontal cortex and anterior cingulate cortex (ACC), along with the left nucleus accumbens and surrounding caudate reliably discriminate trust responses from attraction responses by activating more strongly to attractive face stimuli. If self-expansion or romantic love diminish attraction or attention to alternative partners, then neural activity in this system should be lower to faces images of alternative partners after primes of self-expansion or romantic love.

## Method

### Participants

Couples were recruited via flyers posted on and off Stony Brook University’s campus, emails to listservs and online advertisements on Craigslist and other website advertising the study. Scanned participants were 18 individuals (12 males and 6 females) in heterosexual long-term relationships (at least 2 years). Partners of each of these 18 also participated in the development of the stimuli. Participants ranged in age from 19 to 42 years (*M* = 24.10, SD = 6.17). Overall, couples’ relationship length ranged from 2 to 24 years (*M* = 3.89, *SD* = 4.84), and 3 of the 18 couples were either engaged or married, 3 were dating and living together, and 12 were dating and living separately. Three of the 18 participants who were scanned preferred their left hand.

When participants contacted us expressing interest in the study, we scheduled a phone screening session. During screening, we verified that couples met all inclusion criteria and made sure that one partner was willing and able to enter the scanner safely (no history of claustrophobia, head trauma, drug use, embedded metals, etc.). If both members of the couple were safe to enter the scanner, we allowed the couple to decide who would be in the scanner and who would be outside. Participants were made aware that the study consisted of two in-person sessions (where they would both have to come in to Stony Brook University), with the scan taking place during the second session. Participants were initially told that the study focused on general processes in romantic relationships and at the conclusion of the study, were fully debriefed on the specific hypotheses under investigation.

### Session I (Pre-scanning)

The fMRI participant (who later was scanned) and his or her partner (who was not scanned) were asked to identify together some ways in which life with one another is exciting, novel, and challenging, and to give specific examples of such experiences (e.g., things they’ve done together, joint projects). They were then asked the same thing for times in which they felt strong feelings of love for one another (e.g., the day they got engaged or married). They were also asked to describe some times in which they engage in mundane, routine activities together (e.g., grocery shopping, doing laundry). The experimenter then identified specific instances and experiences related to each of the three categories (i.e. self-expansion, love, and neutral/control).

The fMRI participant and the partner were then separated. The partner was then taken into a separate room where he or she was asked to describe the experiences mentioned above in detail as if they were speaking to their partner (the fMRI participant) and recounting the experiences, and specifically to describe how they felt (and how their partner, the fMRI participant, said they felt) during each of the experiences. While they were describing these experiences, they were videotaped and they were aware of the recording. These video clips were then used (in the scanner in session 2) to prime self-expansion, love, and a neutral/control condition. After editing, a total of two video clips (38 s each) were generated for each of the three conditions. Both partners were unaware as to the exact purpose of the video clips in the study and how they would be used.

While the partner was being interviewed, the fMRI participant completed the following questionnaires in a separate room: Experiences in Close Relationships Scale (Brennan et al., 1998), the Relationship Assessment Scale, a short version of the Passionate Love Scale (Hatfield and Rapson, 1987), a short version of the Self-Expansion Questionnaire (Lewandowski and Aron, 2002), a short version of the Investment Model Commitment Scale (Rusbult, 1983), and the Inclusion of Other in the Self Scale (Aron et al., 1992). When the interview was complete, the fMRI participant (who was later scanned) also completed these questionnaires in a separate room.

### Session II (Scanning)

Approximately 2 weeks later, the fMRI participants and their partners returned to the lab to complete the second session. The second session was scheduled approximately 2 weeks later to allow time for editing of the video clips and to reduce the likelihood of carryover effects from the first session. The participant had an fMRI (functional magnetic resonance imaging) scan of their brain completed at Stony Brook University’s Social, Cognitive, and Affective Neuroscience Center. The partner waited in a separate room within the same building.

**FIGURE 2:**
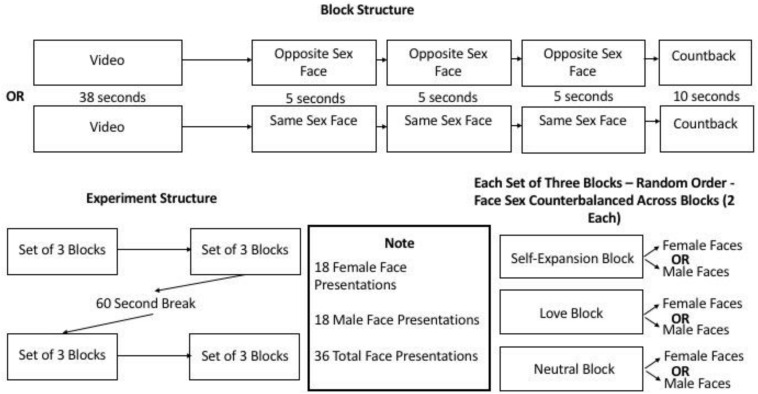
The top of figure 2 diagrams the trial structure of each block. The bottom left of the figure is a diagram of the overall experimental structure broken down by sets of three trials. The bottom right of the figure shows how each set of three blocks includes a block of each video type followed by a set of either female or male faces depending on randomization and counterbalancing. The bottom middle of the figure includes a note on the number of face presentations of each sex.

During the scan, the participant first viewed 10 s of general instructions, then passively viewed the following stimuli:

a. 12 blocks of videos were followed by 3 photos of faces (sometimes all male, sometimes all female, but never mixed). These photos were successfully used in past studies (e.g., Maner et al., 2008; Maner et al., 2009) to measure visual attention to attractive faces. 4 blocks (38 s videos) per video type (self-expansion, love, neutral), 2 followed by male and 2 followed by female faces (5 s presentations each). Each video type was present in each set of three blocks: first three, second three, third three, and fourth three blocks. There were six blocks prior to a break (60 s), and 10 s countback tasks (counting back from a very large number in increments of 7) after each block. See Figure 2 for a diagram.

The Attention to Alternatives Scale and a few brief questions on potential infidelity in their relationship (subjective/objective infidelity measure) were also completed by both partners in the second session post-scanning.

### Data Acquisition and Analysis

Preprocessing and analysis of fMRI images was conducted via FMRIB’s Software Library (FSL) software (Version 5.98^1^; Worsley, 1994). We used FMRIB’s Linear Image Registration Tool, an intra-modal correction algorithm tool (MCFLIRTl; Jenkinson et al., 2002), with slice scan time correction and a high-pass filtering cutoff point of 100 s, removing irrelevant signals. BET (Smith, 2002) brain extraction was employed to remove non-brain material, and smoothing involved a 5-mm full width at half minimum Gaussian kernel. We registered images to the Montreal Neurological Institute (MNI) space by FLIRT (Jenkinson et al., 2002).

#### Primary fMRI Data Analysis

Primary analysis was carried out using FEAT (FMRI Expert Analysis Tool) Version 5.98, part of FSL (FMRIB’s Software Library see text foot note 1) and time-series analysis by FILM (Worsley, 2001). At the first level of analysis a contrast of opposite-sex image minus same-sex image (OS-SS) was used as the primary contrast. Additional contrasts compared OS-SS between video prime conditions (self-expansion, love, and neutral) contrasting each condition with each other condition. A group level analysis was then employed using FSL Randomize (Winkler et al., 2014), which uses non-parametric methods resulting in better control of type I error rates (Eklund et al., 2016; see also Zhang et al., 2012 for an analysis of the relative benefits of non-parametric approaches). Specifically, we employed threshold free cluster enhancement (TFCE; Smith and Nichols, 2009) to identify significant voxels in each contrast. This approach is more conservative and effective at controlling type 1 errors than cluster-wise correction methods, producing voxel-wise outputs that enhance “cluster-like” structures.

Each contrast was tested via both whole brain correction and small volume correction (SVC). Our SVC used three small volume masks based on meta-analyses of neural responses to facial attractiveness (Mende-Siedlecki et al., 2013). This meta-analysis indicated three primary regions of interest in which target facial attractiveness led to greater activity than target trustworthiness across several studies. The regions include the anterior cingulate cortex (ACC; 0, 36, 6), portions of medial prefrontal cortex (mPFC; 4, 44, −6), and left striatum specifically caudate and nucleus accumbens (NAcc; −8, 14, −8). We constructed three masks for ACC, mPFC, and left striate using the Harvard-Oxford cortical structural atlas and the Harvard-Oxford subcortical structural atlas. The ACC mask included all voxels (1807 voxels) identified as at least 50% likely to reside in the cingulate gyrus, anterior division. We included ventral paracingulate and medial prefrontal cortex from the Harvard-Oxford cortical structural atlas in the mPFC mask. This was driven by the peak mPFC coordinates (4, 44, −6) from Mende-Siedlecki et al. (2013) falling outside and dorsally to the mPFC, as defined by the Harvard-Oxford Cortical Structural Atlas, in the ventral paracingulate gyrus. The mPFC mask included all voxels (981 voxels) 50% likely to reside in paracingulate gyrus and frontal medial cortex between MNI z-coordinates −20 and 4. The left striate mask included all voxels (533 voxels) at least 50% likely to reside in the left caudate or NAcc.

#### Secondary fMRI Analyses

Additional exploratory analyses were conducted on BOLD response during video watching. For these analyses the processing stream proceeded the same as for the face images, with first level contrast of love video – self-expansion video, love video – neutral video, self-expansion video – love video, self-expansion video – neutral video, neutral video – love video, and neutral video – self-expansion video.

#### Meta-Analysis Based ROI Analyses

To further substantiate the small volume corrected analysis, we also created 9 mm^3^ masks around each peak voxel identified as responding to facial attractiveness more than trustworthiness in Mende-Siedlecki et al. (2013) meta-analysis (i.e. ACC; 0, 36, 6; mPFC; 4, 44, −6; and NAcc; −8, 14, −8). This involved getting the mean percent signal change from all voxels within each mask for the opposite sex minus same sex face contrast and conducting one-tailed paired-samples *t*-tests on the comparison between each of the three conditions within each ROI. Based on theory and findings regarding derogation of alternatives, we predicted lower BOLD response in the self-expansion condition relative to both the love and neutral conditions in all three ROIs, and lower BOLD response in the love condition relative to the neutral condition. These analyses were conducted in JASP (JASP Team, 2019).

## Results

### Main Effects of Prime Condition

No significant main effects in the focal contrasts were detected in the whole brain corrected analysis. Small volume correction revealed main effects in the ACC and mPFC in the love prime minus self-expansion and neutral prime minus self-expansion conditions. No significant effects were found in any other contrast: self-expansion minus love, self-expansion minus neutral, neutral minus love, or love minus neutral.

#### Love Prime Minus Self-Expansion Prime Contrast

**FIGURE 3:**
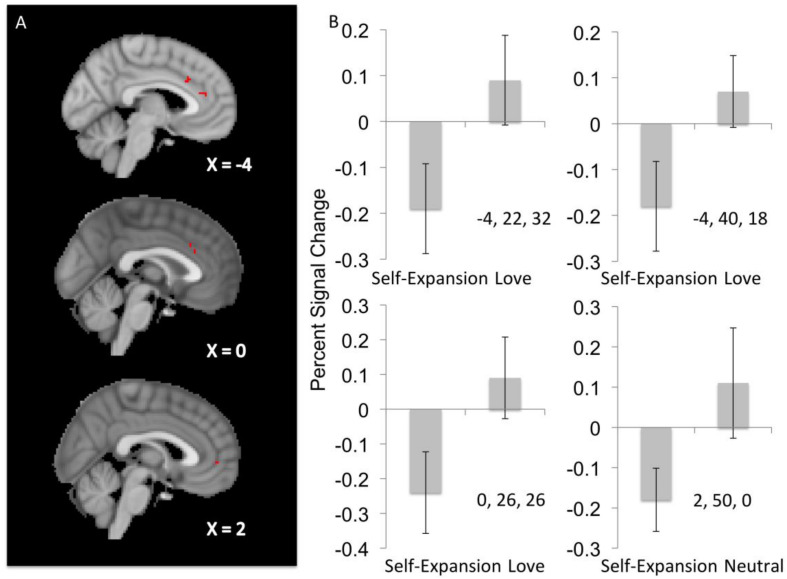
**(A)** Displays sagittal slices with the locations of significant clusters of activity in the Love-Self-Expansion contrast (top two slices) and Neutral-Self-Expansion contrast (bottom slice) during opposite sex face perception (relative to same sex face perception). **(B)** Shows four bar graphs of mean percent signal change, with 95% confidence intervals, in the relevant prime conditions (i.e. Self-Expansion and Love or Self-Expansion and Neutral) during opposite sex face perception (relative to same sex face perception). MNI coordinates are placed in the lower right quadrant of each graph to indicate the ROI being displayed.

Three significant clusters of activity were detected in ACC indicating increased activity to opposite sex faces relative to same sex faces after a romantic love prime relative to after a self-expansion prime. The largest cluster (ACC1; −4, 22, 32) peaked most dorsally relative to the smallest cluster (ACC3; 0, 26, 25), which was dorsal to the middle cluster (ACC2; −4, 40, 18). Follow up analyses indicate that whereas ACC was more active to opposite sex attractive faces after the love prime, it was less active following the self-expansion prime (see Figure 3 and Table 1 for primary results, and Table 2 for estimates of effect size and effect size 95% confidence intervals for each contrast).

**TABLE 1:** Significant clusters of activity for the main effects of prime on perception of opposite sex vs. same sex faces.

Structural location	Number of voxels	*Z*-max	*X*	*Y*	*Z*
Love – self expansion					
dACC	12	3.42	−4	22	32
dACC	5	3.77	−4	40	18
dACC	3	3.26	0	26	26
Neutral – self-expansion					
mPFC	4	3.75	2	50	0

**TABLE 2:** Cohen’s *d* and 95% confidence intervals for each contrast exploring the main effects of prime on perception of opposite sex vs. same sex faces within each functional region of interest.

			95% CI of Cohen’s *d*	
			
ROI	Contrast	Cohen’s *d*	Lower bound	Upper bound
dACC1	Self-expansion – love	–0.792	–1.315	–0.251
	Self-expansion – neutral	–0.663	–1.167	–0.143
	Love – neutral	0.1	–0.364	0.562
dACC2	Self-expansion – love	–0.861	–1.396	–0.308
	Self-expansion – neutral	–0.452	–0.932	0.04
	Love – neutral	0.162	–0.306	0.624
dACC3	Self-expansion – love	–0.767	–1.286	–0.23
	Self-expansion – neutral	–0.495	–0.979	0.002
	Love – neutral	0.148	–0.319	0.61
mPFC	Self-expansion – love	–0.489	–0.973	0.007
	Self-expansion – neutral	–0.875	–1.412	–0.319
	Love – neutral	–0.078	–0.54	0.386

#### Neutral Prime Minus Self-Expansion Prime

Activity to opposite sex faces relative to same sex faces was also diminished after the self-expansion prime relative to the neutral prime in the mPFC (2, 50, 0). Similar to the effect of self-expansion in ACC it appears that mPFC responses to faces were decreased as a function of the self-expansion prime, whereas they were increased in the neutral condition.

### Meta-Analysis Based ROI Analysis Results

To supplement the small volume corrected analyses, we also conducted analyses on mean percent signal change in each condition extracted from 9 mm^3^ masks focused on the peak coordinates from Mende-Siedlecki et al. (2013). Given that these coordinates were derived independently from the data, this approach strengthens our interpretation if it provides agreement with SVC analyses. All analyses were one-tailed paired samples *t*-tests. Because each of these regions of interest are associated specifically with increased responses to attractive faces, this approach provides a relatively strong test of the specific hypotheses that self-expansion and love may lead to derogation of alternatives by focusing on attraction sensitive neural activity.

In the mPFC ROI, mean percent signal change in the self-expansion condition (*M* = −0.098, SE = 3.85) was not significantly less than in the love condition (*M* = −0.534, SE = 4.82), *t*(17) = −1.344, *p* = 0.098, *d* = −0.317, nor was percent signal change lower in the love condition than in the neutral condition (*M* = 1.45, SE = 2.75), *t*(17) = −0.249, *p* = 0.403, *d* = −0.059. There was a significantly less percent signal change in the self-expansion condition relative to the neutral condition, *t*(17) = −2.054, *p* = 0.028, *d* = −0.484. This indicates less neural activity to attractive opposite sex faces in a region associated with responding to attractive faces after the self-expansion manipulation when compared to a neutral condition, supporting one of the primary hypotheses in this ROI.

In the ACC ROI, mean percent signal change in the self-expansion condition (*M* = −7.64, SE = 2.43) was significantly less than in the love condition (*M* = 2.70, SE = 3.98), *t*(17) = −2.264, *p* = 0.018, *d* = −0.534. There was also significantly less percent signal change in the self-expansion condition relative to the neutral condition (*M* = 1.18, SE = 4.00), *t*(17) = −1.819, *p* = 0.043, *d* = −0.429. This indicates less neural activity to attractive opposite sex faces in a region associated with responding to attractive faces after the self-expansion manipulation when compared to a neutral. Percent signal change was not significantly lower in the love condition relative to the neutral condition, *t*(17) = −0.242, *p* = 0.594, *d* = 0.057.

In the NAcc ROI, mean percent signal change in the self-expansion condition (*M* = −3.35, SE = 2.75) was not significantly different than in the love condition (*M* = 2.18, SE = 2.75), *t*(17) = −1.111, *p* = 0.141, *d* = −0.262, and not significantly different than in the neutral condition (*M* = −0.142, SE = 3.09), *t*(17) = −0.934, *p* = 0.182, *d* = −0.220. Percent signal change was also not significantly lower in the love condition than in the neutral condition, *t*(17) = 0.423, *p* = 0.661, *d* = −0.100.

### Correlations With Inclusion of Other in the Self

**FIGURE 4:**
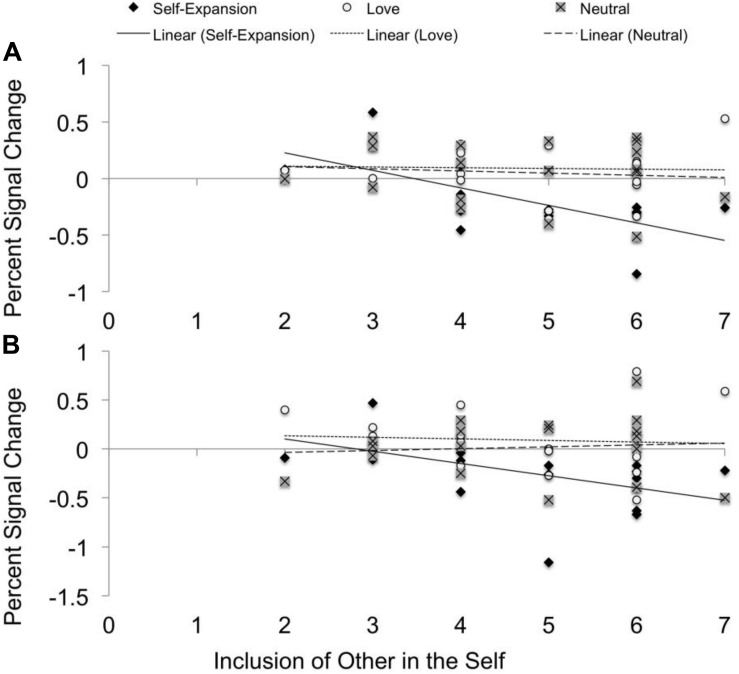
Each scatterplot shows the correlation between percent signal change in each prime condition during opposite sex face perception (relative to same sex face perception) and self-report of relationship closeness using the IOS. The self-expansion condition is indicated by diamonds and an unbroken fit line, the love condition is indicated by circles with a dotted fit line, and the neutral condition is indicated by boxed x’s and a dashed fit line. Scatterplot **(A)** includes the average percent signal change across all voxels in the dACC cluster peaking at MNI coordinates –4, 22, 32. Scatterplot **(B)** includes the average percent signal change across all voxels in the dACC cluster peaking at MNI coordinates 0, 26, 26.

Correlations between IOS and neural response in ACC ROIs indicated a significant negative correlation between IOS and percent signal change in the opposite sex face minus same-sex face contrast in the self-expansion prime condition, ACC1, *r*(18) = −0.68, *p* = 0.002, ACC3 *r*(18) = −0.50, *p* = 0.037, indicating that those who reported greater inclusion of their partner in the self during the scanning session also had decreased ACC neural response to opposite sex faces (relative to same sex faces) during the self-expansion condition (see Figure 4). No significant correlations were found for the love condition, nor for the neutral condition.

### Secondary Analyses

Blood oxygenation level dependent response during presentation of the different video types was contrasted across all possible paired comparisons. We found regions of enhanced activation to the love videos relative to both the neutral and self-expansion videos. We also found regions of enhanced activation to the neutral video relative to the love video. No significant effects emerged in the other contrasts: neutral minus self-expansion, self-expansion minus love, and self-expansion minus neutral.

#### Love Minus Neutral Video

Several significant clusters of activity indicated regions in which activity was greater during the love video relative to the neutral video. Clusters peaked in the medial frontal pole, bilateral superior temporal gyrus, right thalamus, occipital cortex, hippocampal, parahippocampal, and temporal fusiform regions. These activations are consistent with heightened visual attention to social stimuli, indicating that the love videos may have enhanced activity in networks associated with social perception processes (see Table 3 for coordinate and cluster details).

**TABLE 3:** Significant clusters of activity for the main effects of video type.

Structural location	Voxels	*Z*-max	**X**	**Y**	**Z**
Love minus neutral					
Medial frontal pole	848	5.18	0	64	2
Sup. temporal gyrus	780	5.73	−56	−14	−4
Sup. temporal gyrus	363	4.66	50	−2	−22
Thalamus	85	5.29	2	−6	6
Occipital pole	84	4.82	2	−90	0
Putamen	33	4.2	−20	16	−4
Parahippocampal	12	5.73	−16	−26	−12
Occitpital pole	8	5.25	−26	−98	−2
Temporal fusiform	8	4.44	40	−10	−24
Occitpital pole	4	4.5	28	−98	−10
Hippocampus	3	5.24	−38	−20	−12
Love minus self-expansion					
Sup. occipital	2	6.91	−36	−74	56
Sup. occipital	1	6.67	−34	−78	54
Neutral minus love					
Posterior cingulate	4	6.86	6	−26	22
Posterior cingulate	1	7.01	−6	−30	20

#### Love Minus Self-Expansion Video

Two significant clusters of activity indicated regions in which activity was greater during the love video relative to the self-expansion video. Both clusters peaked in occipital cortex reinforcing the possibility that visual attention was heightened during the love videos (see Table 3).

#### Neutral Minus Love Video

Two significant clusters of activity indicated regions in which activity was greater during the neutral video relative to the love video. Both clusters peaked in the posterior cingulate cortex. This may indicate greater default mode network activity during the neutral video, indicating increased internal attention (see Table 3).

## Experiment 2 Discussion

We found neural activity in regions associated with perceiving attractive faces of the opposite sex to be diminished in the self-expansion condition relative to the romantic love and neutral conditions. This diminished activity was detected in both anterior cingulate and medial prefrontal cortices. The anterior cingulate is a core hub in the salience network (Sridharan et al., 2008), which involves switching from internal attention to external attention. This network is commonly activated by emotionally evocative stimuli, surprising events, cognitive errors, and other occurrences that require the focusing of executive resources and attention on the current context. It is unsurprising that viewing an attractive member of one’s preferred sex would activate this network given its role in processing of motivationally significant stimuli. The medial prefrontal cortex is frequently involved in processing emotional information and reward, and is part of the ventral dopamine pathway (Berridge, 2012) that signals incentive salience, or “wanting”. The subsection of the mPFC found in this study is associated with both affective and decision making processes (de la Vega et al., 2016), and may be critical for using affective information in the service of decision making and other cognitive functions.

The modulation of the aforementioned regions suggests that priming for self-expansion may lead to reduced interest in alternatives by diminishing the perceived attractiveness of potential alternatives. Participants who reported greater IOS after the self-expansion manipulation also had decreased attraction-correlated neural response to opposite sex faces (relative to same sex faces). Each of these findings support the hypothesis that self-expansion might promote derogation of alternatives by altering the incentive salience of alternative faces. Notably, the differences in activity were primarily found in medial prefrontal regions strongly associated with reward processing generally, and interpersonal attraction specifically. Whereas it is possible that these differences are driven by attentional factors, the primary impact of self-expansion on attraction to alternatives seems to occur in regions more strongly associated with motivation and evaluation. Furthermore, this is the first time to our knowledge that this novel procedure to manipulate self-expansion has been used in research.

There are important caveats to these conclusions. First the main effects of prime type on the neural response to attractive opposite sex faces were not robust to whole brain corrections. This likely indicates that the current study is somewhat underpowered and increases the probability that the findings are a type I error. This concern is somewhat mitigated by the use of data independent ROI analyses, and data independent correlations between inclusion of other in the self (an indicator of previous relationship based self-expansion) and activity in the ROIs, but not sufficiently that readers should come away certain that the effects will replicate. Future studies should replicate this finding with larger samples to verify and extend the current findings. Second, although we selectively looked in regions that are well demonstrated to respond to the perception of attractive faces, these regions are also active in many other kinds of contexts and respond to diverse stimuli. Therefore, given the known problems with reverse inference (Poldrack, 2006), such conclusions about what the neural response might mean should be taken with appropriate caution.

Notably, no differences were found between the romantic love and neutral conditions. Whereas previous research has shown behavioral evidence for diminished attention to alternatives in conditions enhancing feelings of romantic love, we found no evidence for diminished attention to alternatives in the love condition relative to the neutral condition. This may be due to methodological differences between the studies, slower memory consolidation processes that fMRI was unable to detect that led to the effects in behavioral studies, or due to inadequate power to detect any effects.

In addition to the primary findings, we also found differences in BOLD response to videos of different types. Love videos evoked greater BOLD response relative to neutral videos in large portions of the brain associated with the processing of social stimuli, such as the superior temporal lobe and medial prefrontal cortex. Additional activity indicated greater involvement of regions associated with long term memory in hippocampal and parahippocampal regions, heightened visual attention in the occipital cortex and fusiform gyrus, and subcortical regions associated with motivation and affect such as the putamen. Most of this heightened activity was not seen when compared to the self-expansion video. The only region with greater BOLD response during the love videos relative to the self-expansion videos included occipital cortex. These findings tentatively suggest that love videos induce greater psychological engagement relative to neutral videos, but only increase visual attention relative to self-expansion.

One potential for future research involves an investigation of how relational self-expansion may be linked to implicit (as measured by fMRI) evaluations of one’s partner and implicit evaluations of alternatives. Perhaps the most promising future direction for research following up on these effects would include a way to overcome the reverse inference problem by identifying activity that is attraction specific. This may be possible with the right types of analytic methods and sufficient neuroimaging groundwork. For example, multivoxel pattern analysis has been successfully used to identify neural activity specifically associated with physical pain (Wager et al., 2013). If one could identify attraction specific activity, then one could apply similar methods used in this study to verify that the reductions in activity observed are specifically a function of diminished attraction to potential alternatives.

## Conclusion

In conclusion, infidelity is a widespread phenomenon that can greatly affect the welfare of individuals, their partners, and families. And more generally the role of relationship alternatives is central to theories of relationship commitment. As suggested by the findings of Experiment 1, identifying circumstances under which different kinds of alternatives are seen as most desirable may be an important factor in helping individuals avoid the temptation of attractive alternatives. The self-expansion model represents a novel approach that may elucidate some of the factors that determine how strongly people cognitively process – and experience the pull of – attractive relationship alternatives.

Experiment 2 is tentatively suggestive that self-expansion primes promote derogation of alternative partners by reducing their perceived attractiveness directly, whereas romantic love primes do not. This reduction in attraction to alternatives appears to occur in the processing of attractiveness per se, not necessarily via decreased attention to the alternatives. This may be because the current relationship is bolstered by feelings of self-expansion diminishing the relative attractiveness and therefore the incentive salience of alternative partners.

These findings, as well as the methodological innovations, both Experiment 1 and Experiment 2, suggest promising future directions for applying the self-expansion model to research on infidelity and pair bonding. Whereas the importance of the findings themselves offer an initial glimpse into the causal effect of self-expansion on attraction to alternatives, the methodological innovation related to manipulating self-expansion may be even more important and should open up new avenues for future research.

## Data Availability Statement

The datasets generated for this study are available on request to the corresponding author.

## Ethics Statement

The studies involving human participants were reviewed and approved by the Stony Brook IRB. The patients/participants provided their written informed consent to participate in this study.

## Author Contributions

IT collected the data, designed the study, analyzed the data, and wrote the manuscript. LB analyzed the data, wrote the manuscript, and interpreted of the fMRI data. AA analyzed the data, wrote the manuscript, and designed the study.

## Conflict of Interest

The authors declare that the research was conducted in the absence of any commercial or financial relationships that could be construed as a potential conflict of interest.

## Footnotes

1 www.fmrib.ox.ac.uk/fsl
